# Periodontitis and GLP-1 pathways: a new frontier in oral-systemic health connections —a scoping review

**DOI:** 10.3389/fcdhc.2025.1679511

**Published:** 2025-11-11

**Authors:** Natalie Jeong, Lin-Hsin Chuang, Yolanda Ho

**Affiliations:** Department of Periodontology, School of Dental Medicine, Tufts University, Boston, MA, United States

**Keywords:** GLP-1, periodontitis, oral-systemic association, diabetes mellitus, obesity

## Abstract

Periodontitis, a chronic inflammatory disease of the periodontium, has well-established links to systemic metabolic conditions, particularly diabetes and obesity. Recent research suggests a novel interaction between periodontitis and glucagon-like peptide-1 (GLP-1) pathways, both of which regulate glucose metabolism and inflammation. In this review, we examine the potential bidirectional relationships between periodontitis and GLP-1 signaling and evaluate the therapeutic implications of GLP-1 receptor agonists (GLP-1 RAs) in periodontal disease. A systematic search of PubMed, Embase, and the Cochrane Library identified 52 studies published between 1990 and 2025, ranging from *in vitro* and animal studies to human clinical and observational research. Findings indicate a multifaceted relationship between GLP-1 pathways and periodontal disease. Periodontitis may impair GLP-1 signaling and exacerbate glucotoxicity and lipotoxicity in individuals with diabetes or obesity. Several periodontopathic bacteria, notably *Porphyromonas gingivalis*, produce DPP-4-like enzymes that degrade GLP-1 and potentially disrupt glucose regulation. GLP-1 RAs, such as liraglutide and exendin-4, demonstrated anti-inflammatory, osteoprotective, and regenerative effects in preclinical models. Additionally, studies identified host and microbial DPP-4 activity as key mechanistic links between periodontal inflammation and systemic insulin resistance. This review highlights a novel and clinically relevant intersection between periodontitis and GLP-1 biology. GLP-1 RAs and DPP-4 inhibitors may offer dual benefits for metabolic control and periodontal health. Further research is needed to define delivery strategies, assess efficacy across patient populations, and explore the therapeutic targeting of DPP-4 activity in both host and microbial contexts.

## Introduction

Periodontitis, a chronic inflammatory disease affecting the supporting structures of the teeth—including the gingiva, periodontal ligament, and alveolar bone—is one of the leading causes of tooth loss worldwide. It is increasingly recognized for its systemic health implications. Its pathogenesis involves dysbiosis that triggers a persistent host inflammatory response, leading to destruction of periodontal tissues, including the alveolar bone. Beyond local effects, periodontitis is strongly associated with systemic conditions such as cardiovascular disease and obesity and it has an established bidirectional relationship with diabetes, underscoring the interconnectedness of oral and systemic health (1, 2).

A previous scoping review has highlighted emerging evidence of a potential interplay between periodontitis and the incretin hormone glucagon-like peptide-1 (GLP-1), a key regulator of glucose metabolism with anti-inflammatory properties. The novelty of this review is its focus on GLP-1 receptor agonists (GLP-1 RAs), widely used to treat type 2 diabetes and obesity. GLP-1 RAs have shown promise in reducing systemic inflammation and promoting tissue regeneration (3, 4). Because type 2 diabetes and obesity share similar GLP-1 pathways of metabolic dysregulation, this review considers how these pathways may be leveraged in managing inflammatory conditions such as periodontitis.

A key aspect of this relationship is the role of dipeptidyl peptidase-4 (DPP-4), the enzyme responsible for degrading GLP-1. Notably, certain periodontopathic bacteria, such as *Porphyromonas gingivalis*, exhibit DPP-4–like enzymatic activity, which may disrupt GLP-1 signaling and glucose homeostasis (5–7). This mechanism suggests a bidirectional interaction between periodontitis and GLP-1 pathways, in which periodontal inflammation could exacerbate systemic metabolic dysfunction and vice versa.

Preclinical studies have further explored the therapeutic potential of GLP-1 RAs, such as liraglutide, in mitigating periodontitis. These agents have demonstrated anti-inflammatory effects and promoted bone regeneration in experimental models, suggesting a dual benefit for managing both metabolic and periodontal health (8–10). However, the direct mechanistic links and clinical relevance of these findings remain under investigation.

Given the shared inflammatory and metabolic pathways underlying periodontitis, diabetes, and obesity, understanding the interactions between these conditions and GLP-1 pathways is critical. Unlike prior reviews (e.g., 11), which emphasized the periodontitis–diabetes axis, this review takes a broader perspective that integrates obesity-related evidence and highlights therapeutic implications across both populations.

This review addresses the following four specific objectives applied throughout the manuscript:

1. To evaluate population-specific variations in periodontal outcomes associated with GLP-1 RAs among individuals with type 2 diabetes.
2. To evaluate population-specific variations in periodontal outcomes associated with GLP-1 RAs among individuals with obesity but without diabetes.
3. To examine the role of DPP-4 activity in periodontitis and its interaction with GLP-1 signaling.
4. To synthesize evidence on the therapeutic effects of GLP-1 RAs—including anti-inflammatory, bone-preserving, and tissue-regenerative actions—in periodontal disease.

## Materials and methods

This scoping review was conducted to synthesize and evaluate existing evidence on the interplay between GLP-1 RAs and periodontitis. The review aimed to investigate the therapeutic potential and underlying mechanisms of GLP-1 RAs in the context of periodontal health, focusing on outcomes in individuals with type 2 diabetes, those with obesity but without diabetes, and mixed populations. The methodological framework was guided by the five-step process described by Arksey and O’Malley, with refinements, and was further informed by recommendations by Levac et al. to enhance rigor and transparency in scoping reviews.

### Identification of the research question

The central research question was the following:

*“What is the role of GLP-1 pathways in linking metabolic dysregulation and periodontal disease, and how might GLP-1 receptor agonists contribute to periodontal therapy across diabetic and obese populations?”*

This question emerged from an exploratory literature review that revealed emerging evidence of GLP-1 RAs modulating inflammatory pathways, bone metabolism, and periodontal tissue healing.

### Literature search strategy

A comprehensive literature search was performed using PubMed, Embase, and the Cochrane Library to identify relevant studies. The search strategy incorporated combinations of Medical Subject Headings (MeSH) terms and keywords, including the following:

- Interventions: “semaglutide, “ “Ozempic, “ “Rybelsus, “ “Wegovy, “ “GLP-1 receptor agonist, “ “Glucagon-like peptide-1, “ “dulaglutide, “ “exenatide, “ “liraglutide, “ “lixisenatide, “ “DPP-4, “ “Dipeptidyl peptidase-4, “ “linagliptin, “ “sitagliptin, “ “alogliptin.”
- Disease context: “periodontitis, “ “periodontal disease.”

These agents and trade names were chosen because they represent widely studied and clinically approved GLP-1 RAs and DPP-4 inhibitors for diabetes and obesity management. Including both generic names and trade names ensured a comprehensive capture of relevant studies across clinical and translational research.

The search included peer-reviewed studies published in English between January 1990 and January 2025. Bibliographies of selected articles were also manually screened for additional relevant studies.

### Study selection

Inclusion criteria were the following:

- Investigated the effects of GLP-1 RAs or DPP-4 inhibitors on periodontitis or related biological pathways.
- Examined outcomes such as periodontal inflammation, bone metabolism, tissue regeneration, or systemic effects of periodontitis.
- Included diabetic populations, obese populations, or otherwise healthy controls.
- Were original peer-reviewed articles, encompassing randomized controlled trials, observational studies, *in vitro* experiments, or scoping and systematic reviews where relevant.

Exclusion criteria were the following:

- Non-English publications.
- Studies without accessible full texts.
- Articles not directly evaluating the interaction between GLP-1 RAs and periodontal health.

Screening was performed in two phases: title and abstract review, followed by full-text review for eligibility.

### Data charting and synthesis

Data from the selected studies were extracted and charted using Microsoft Excel (Microsoft Corporation, United States). Key variables included:

- Study characteristics: author(s), publication year, study design, and population demographics.
- Methodological details: interventions (e.g., GLP-1 RAs or DPP-4 inhibitors), outcomes measured, and analytic approaches.
- Key findings: effects of GLP-1 RAs on inflammation, bone preservation, and periodontal regeneration, stratified by population (diabetic, obese, or mixed).

Charted data were synthesized into descriptive summaries to highlight emerging patterns and gaps in the literature.

### Reporting and analysis

The findings are presented as a narrative synthesis organized around four objectives:

1. Impact of Periodontitis on GLP-1 Levels and Glucose Metabolism in Diabetes
2. Interplay Among Periodontitis, GLP-1 Pathways, and Dyslipidemia in Obesity
3. Role of DPP-4, Periodontopathic Bacteria, and Molecular Pathways in Periodontitis
4. Therapeutic Potential of GLP-1 Receptor Agonists in Periodontal Inflammation and Regeneration

The review process and study selection followed PRISMA-ScR guidelines, with a revised PRISMA flow diagram provided.

## Results

### Search results and study selection

**Figure 1:**
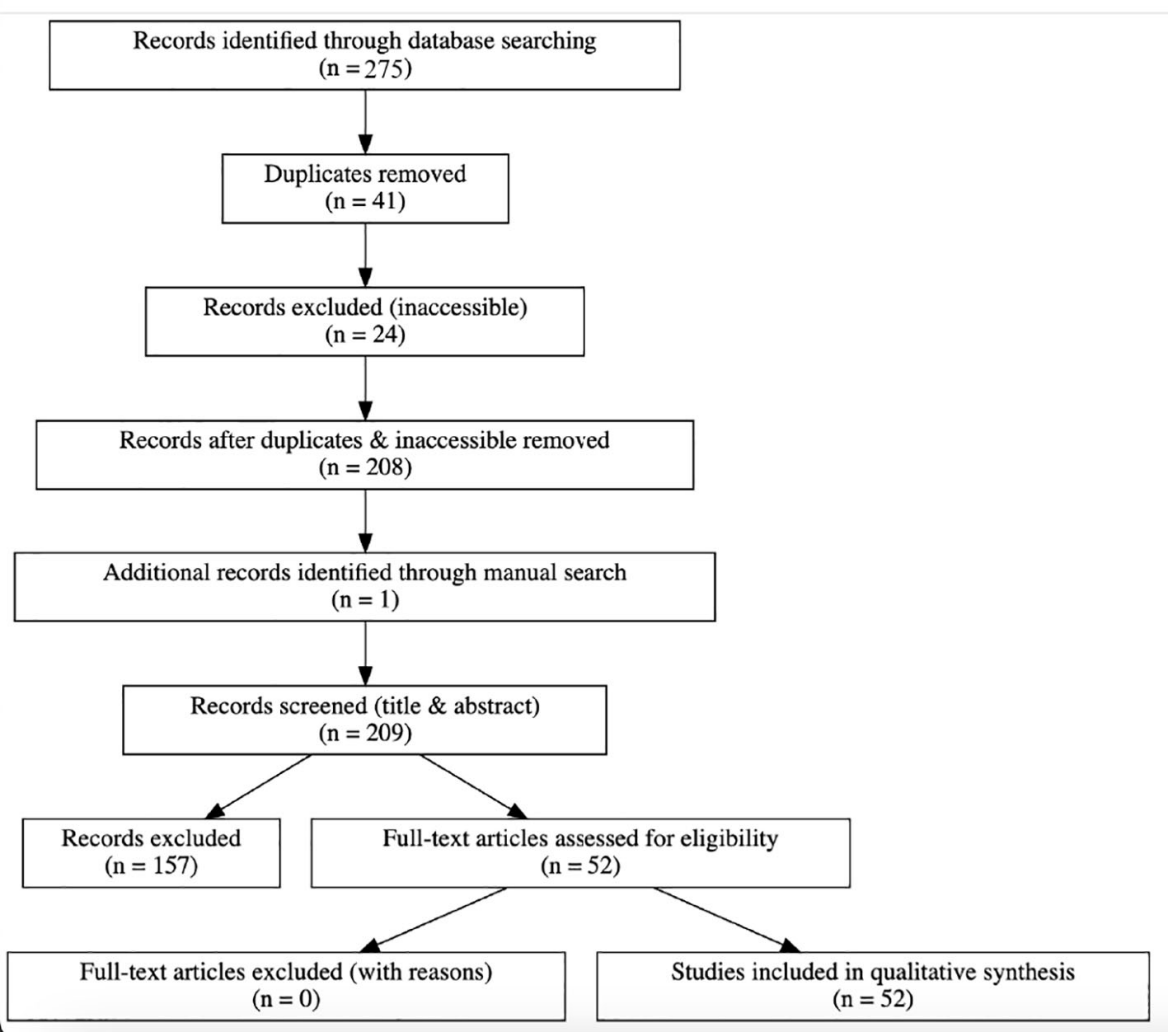
PRISMA flow diagram.

A total of 275 records were identified through electronic database searches (PubMed, Embase, and Cochrane). After removing 41 duplicates and excluding 24 inaccessible articles, 208 unique records remained for title and abstract screening. One additional study was identified through manual searching. After screening and conducting full-text reviews, 52 studies met the inclusion criteria and were included in the final synthesis (**Figure 1**: PRISMA Flow Diagram).

### Thematic synthesis of evidence

#### Impact of periodontitis on GLP-1 levels and glucose metabolism in diabetes

Eight studies investigated how periodontitis influences GLP-1 activity, glucose metabolism, and related pathways under glucotoxic and lipotoxic conditions, particularly in the context of diabetes (**Table 1**).

**Table 1:** Impact of periodontitis on GLP-1 levels and glucose metabolism in diabetes.

Author(s)	Year	Type of publication	Population	Intervention	Outcomes measured	Key findings	Limitations
Gheonea TC et al. (11)	2024	Scoping Review	N/A	N/A	Role of DPP-4 and GLP-1 in periodontitis-diabetes link	Suggests DPP-4 inhibitors and GLP-1 agonists may benefit periodontal and glycemic outcomes	Limited to existing literature, no experimental data
Sun Y et al. (12)	2024	Review	N/A	N/A	Lipotoxicity in diabetes and periodontitis	Proposes lipotoxicity as a key mechanism linking diabetes and periodontitis	Conceptual framework; lacks empirical evidence
Kang WS et al. (13)	2021	Experimental Study (Animal)	Diabetic rat model	Gemigliptin (DPP-4 inhibitor)	Salivary function, glucose levels	Gemigliptin improves salivary function in diabetic rats	Animal study; lack of human data limits clinical relevance
Wang M et al. (14)	2023	Experimental Study (Cell culture)	Periodontal ligament stem cells	Exendin-4 (GLP-1 receptor agonist)	MAPK and WNT signaling, osteogenic activity	Exendin-4 alleviates osteogenic inhibition in high glucose environment by modulating signaling pathways	*In vitro* study; effects in humans not confirmed
Yang M et al. (15)	2022	Review	N/A	N/A	Therapeutic potential of Liraglutide in diabetes-periodontitis comorbidity	Decreased alveolar bone resorption (reduced RANKL/OPG ratio), improved microvasculature of alveolar bone, reduced periodontal inflammation (IL-6, TNF-a, IL-1b) (p<0.05), upregulated ALP mRNA and Runx2 mRNA in gingival epithelium (impt for osteoblast differentiation).	Theoretical analysis; lacks primary data
Wang Z et al. (16)	2020	*In vitro* study	Human periodontal ligament stem cells (hPDLSCs)	GLP-1	PKCβ2 phosphorylation, osteogenic differentiation	GLP-1 inhibited PKCβ2 phosphorylation, enhancing osteogenic differentiation in an AGE microenvironment	*In vitro* findings; requires *in vivo* validation
Mohamed HG et al. (17)	2015	Cross-sectional Study	Adults with and without Type 2 Diabetes	Measurement of glucoregulatory biomarkers	GCF biomarker levels and periodontitis severity	Chronic periodontitis was linked to altered glucoregulatory biomarkers, highlighting a possible link between periodontitis and metabolic dysregulation	Cross-sectional design limits causal conclusions; potential confounders not fully accounted for
Bajinka O et al. (18)	2023	Review	N/A	Review of gut microbiota pathways	Diabetes mechanisms	Explored the role of gut microbiota in diabetes, including GLP-1 modulation, highlighting potential links to periodontal health	Review article; lacks direct experimental evidence

- Narrative reviews (11, 12, 15) underscored the theoretical link between GLP-1 pathways and the periodontitis–diabetes axis. Gheonea et al. highlighted potential dual benefits of DPP-4 inhibitors and GLP-1 RAs for periodontal and glycemic outcomes. Sun et al. proposed lipotoxicity as a shared mechanism aggravating both diabetes and periodontal inflammation. Yang et al. summarized experimental evidence that liraglutide reduces alveolar bone resorption, improving bone microvasculature, and downregulates inflammatory mediators (IL-6, TNF-α, IL-1β), while enhancing osteoblast differentiation markers (ALP and Runx2).
- Experimental studies demonstrated protective roles of GLP-1 signaling under hyperglycemic stress. Kang et al. (13) showed that the DPP-4 inhibitor gemigliptin improved salivary function and glycemic control in diabetic rats. Wang et al. (14) reported that Exendin-4 alleviated osteogenic inhibition in periodontal ligament stem cells exposed to high glucose via MAPK and WNT pathway modulation. Similarly, Wang et al. (16) found that GLP-1 enhanced osteogenic differentiation in AGE-rich environment by inhibiting PKCβ2 phosphorylation.
- Clinical evidence remains limited. Mohamed et al. (17) reported altered glucoregulatory biomarker levels in gingival crevicular fluid in type 2 diabetes patients with periodontitis, suggesting metabolic dysregulation associated with periodontal inflammation.
- Microbiome-related mechanisms were highlighted by Bajinka et al. (18), who reviewed how gut microbiota-mediated modulation of GLP-1 could link diabetes and periodontal health.

Together, these studies support a bidirectional link between periodontal inflammation and impaired glucose metabolism, mediated partly by GLP-1 pathways.

#### Interplay among periodontitis, GLP-1 pathways, and dyslipidemia in obesity

Five studies examined links among periodontitis, GLP-1 pathways, and dyslipidemia or lipotoxicity in obesity and metabolic syndrome (**Table 2**).

**Table 2:** Interplay among periodontitis, GLP-1 pathways, and dyslipidemia in obesity.

Author(s)	Year	Type of publication	Population	Intervention	Outcomes measured	Key findings	Limitations
Mesa F et al. (19)	2019	Review	N/A	N/A	Mechanisms linking periodontitis and cardiometabolic risk	Identifies inflammatory pathways and immune responses contributing to cardiometabolic risk in periodontitis patients	Lacks primary data; mainly theoretical perspectives
Solini A et al. (20)	2019	Observational Study	Severely obese individuals	N/A	Glucoregulatory hormones, periodontal status	Periodontitis affects glucoregulatory hormones in obese patients	Observational design; no causal relationship proven
Suvan J et al. (21)	2021	Cohort Study	Obese and non-obese individuals with periodontitis	Periodontal treatment	Changes in incretin hormones (GLP-1, GIP), metabolic markers	Periodontal treatment improved incretin hormone levels, with greater effects in obese patients	Observational design; causality cannot be confirmed
Li F et al. (22)	2023	Experimental Study	Specific Pathogen Free (SPF)-grade Wistar rats	Liraglutide administration	Inflammation, oxidative stress (Nrf2/HO-1 pathway)	Liraglutide reduced inflammation and oxidative stress in periodontitis by activating the Nrf2/HO-1 pathway, suggesting therapeutic potential	Animal study; human clinical relevance needs further investigation
Marchetti E et al. (23)	2009	Review	N/A	Analysis of metabolic syndrome and periodontal disease	Systemic health connection	Highlighted the bidirectional relationship between metabolic syndrome and periodontal disease, emphasizing systemic inflammation pathways	Review article; lacks direct experimental data

- Narrative reviews (19, 23) emphasized the systemic burden of chronic low-grade inflammation as a shared feature of periodontal disease and metabolic syndrome, though without primary data.
- Human studies provided preliminary evidence: Solini et al. (20) showed that periodontitis negatively affected incretin hormone profiles in severely obese individuals, whereas Suvan et al. (21) reported that periodontal treatment improved GLP-1 and GIP levels and metabolic markers, with stronger effects in obese patients.
- Animal evidence (22) suggested that liraglutide mitigated periodontal inflammation and oxidative stress by activating the Nrf2/HO-1 pathway, pointing to a mechanistic role for GLP-1 in obesity-related periodontal disease.

These findings suggest that GLP-1 modulation may represent a shared therapeutic pathway for obesity, metabolic dysregulation, and periodontal disease.

#### Role of DPP-4, periodontopathic bacteria, and molecular pathways in periodontitis

Twenty-seven studies have explored the contributions of DPP-4 activity, bacterial proteases, and host–microbe interactions to periodontal pathogenesis (**Table 3**).

**Table 3:** Role of DPP-4, periodontopathic bacteria, and molecular pathways in periodontitis.

Author(s)	Year	Type of publication	Population	Intervention	Outcomes measured	Key findings	Limitations
Elgün S et al. (24)	2000	Observational Study	Patients with periodontal disease	N/A	Salivary enzyme levels (Alanine aminopeptidase, DPP-IV)	Higher enzyme levels associated with periodontal disease	Small sample size; observational design limits causal inference
Aemaimanan P et al. (25)	2009	Observational Study	Chronic periodontitis patients	N/A	Salivary levels of Alanine aminopeptidase and DPP-IV	Elevated levels of these enzymes were associated with chronic periodontitis	Cross-sectional design; cannot establish causality
Ohara-Nemoto Y et al. (26)	2022	*In vitro* study	Bacterial enzymes	N/A	Substrate specificity of bacterial DPP-7	Expanded substrate specificity enables degradation of bioactive peptides	*In vitro* findings; implications for human health not directly established
Ohara-Nemoto Y et al. (5)	2018	Observational Study	Human oral microbiota	N/A	Distribution of DPP4, DPP5, DPP7, and DPP11	Presence of these DPPs is linked to periodontopathic bacteria, suggesting biomarker potential	Microbiota study; functional impact on periodontitis not fully explored
Ohara-Nemoto Y et al. (6)	2017	Experimental Study (*In vitro* and *in vivo*)	Periodontopathic bacteria	N/A	Incretin degradation and blood glucose modulation by bacterial DPP-4	Bacterial DPP-4 degrades incretins, potentially impacting blood glucose levels	*In vitro* and animal model findings; human relevance needs further validation
Jiang Y et al. (27)	2021	Experimental Study (*In vitro*)	Saliva-derived microcosm biofilms	Manipulation to resemble dysbiotic subgingival microbiota	P. ging added to biofilm and noted increase in DPP4 activity and butyric acid production. Biofilm composition and structure	Successfully manipulated biofilms to mimic dysbiotic subgingival microbiota, offering a model for studying periodontal disease	*In vitro* model; may not fully replicate *in vivo* conditions
Rea D et al. (28)	2017	Experimental Study (Crystallography and inhibitor profiling)	Porphyromonas gingivalis DPP-4 enzyme	Inhibitors targeting DPP-4	Enzyme structure, inhibitor efficacy	Crystal structure revealed active sites for DPP-4, aiding in inhibitor design to combat periodontopathogens	Focuses on structural biology; lacks *in vivo* functional analysis
Kennett CN et al. (29)	1996	Histochemical and immunocytochemical study	Human gingival tissue	N/A	Localization of DPP II and DPP IV	DPP II and IV localized in gingival tissues, suggesting roles in periodontal health and disease	Descriptive study; does not assess functional impact
Teshirogi K et al. (30)	2003	Experimental Study	Porphyromonas gingivalis	Monoclonal antibody against DPP IV	Inhibition of DPP IV activity	Monoclonal antibody inhibited DPP IV activity in P. gingivalis, suggesting potential for therapeutic targeting	*In vitro* study; clinical implications require further investigation
Yost S, Duran-Pinedo AE (31)	2018	Experimental Study	Tannerella forsythia	Analysis of DPP IV role	Collagen degradation	DPP IV contributed to collagen breakdown, implicating its role in tissue destruction in periodontitis	Mechanistic study; needs *in vivo* validation
Cox SW et al. (32)	1992	Observational Study	Human gingival tissue and crevicular fluid	None	DPP II and IV activity in periodontitis lesions	Elevated DPP II and IV activities in periodontitis sites, suggesting involvement in disease progression	Cross-sectional study; cannot establish causality
Cox SW, Eley BM (33)	1992	Observational Study	Chronic periodontitis patients	Basic periodontal treatment	Enzymatic activity (DPP IV, cathepsin B/L, elastase, tryptase, trypsin)	Reduction in DPP IV and other protease activities post-treatment, indicating a role in inflammation and tissue breakdown	Limited sample size; observational design limits causal inference
Clais S et al. (34)	2014	Experimental Study	Clinical isolates of P. gingivalis	Analysis of biofilm formation and DPP IV	Pathogenicity and biofilm formation	DPP IV activity was crucial for biofilm formation and virulence in P. gingivalis, suggesting a target for therapeutic intervention	*In vitro* study; clinical relevance needs further exploration
Rea D et al. (35)	2004	Experimental Study	Porphyromonas gingivalis	Expression and crystallographic analysis of DPP IV	Protein structure and activity	Successfully expressed and purified DPP IV from P. gingivalis, providing a basis for structural and functional studies	Preliminary study; functional implications require additional research
Suzuki A et al. (36)	2004	Genetic Association Study	Severe periodontitis patients	Genomic marker analysis	Genetic susceptibility to severe periodontitis	Identified genomic markers potentially associated with increased risk of severe periodontitis	Observational study; requires validation in larger, diverse populations
Kumagai Y et al. (37)	2000	Experimental Study	Porphyromonas gingivalis	Characterization of DPP IV	Enzymatic properties and virulence	DPP IV contributed to virulence through enzymatic activity, supporting its role in periodontal pathogenesis	*In vitro* findings; *in vivo* effects need to be confirmed
Nemoto E et al. (38)	1999	Experimental Study	Human gingival fibroblasts	Cytokine and bacterial stimulation	CD26/DPP IV expression	Cytokines and bacterial components significantly increased CD26/DPP IV expression on gingival fibroblasts, suggesting a role in immune response and inflammation	*In vitro* study; *in vivo* relevance requires further investigation
Kumagai Y et al. (39)	2005	Experimental Study	Porphyromonas gingivalis	Analysis of DPP IV activity	Connective tissue destruction mechanisms	DPP IV contributed to connective tissue destruction through enzymatic activity, implicating it in periodontal pathogenesis	Mechanistic study; clinical implications need validation
Mizutani T et al. (40)	1990	Cross-sectional Study	Human gingival tissue	Measurement of DPP II and IV activity	Enzyme activity in chronic periodontitis	Increased DPP II and IV activity was observed in periodontitis-affected gingiva, suggesting a link to tissue breakdown	Observational design; lacks longitudinal data to determine causality
Eley BM, Cox SW (41)	1992	Longitudinal Study	Chronic periodontitis patients	Comparison of enzyme activities pre- and post-surgery	Protease activity in GCF	Elevated DPP IV-like activity was detected in GCF, which decreased post-surgery, indicating its involvement in periodontal inflammation and healing	Limited sample size; other inflammatory markers not assessed
Kennett CN et al. (42)	1997	Experimental Study	Gingival crevicular fluid samples	Analysis of proteases and inhibitors	Cellular contribution to protease activity	Host tissue proteases, including DPP IV, were significant contributors to GCF activity, highlighting their role in periodontal disease progression	Focused on *in vitro* enzymatic activity; *in vivo* dynamics require further exploration
Kumagai Y et al. (43)	2003	Experimental Study	Porphyromonas gingivalis	Analysis of DPP IV peptidase activity	Virulence and pathogenicity	DPP IV peptidase activity was crucial for virulence but not solely sufficient, suggesting other factors contribute to P. gingivalis pathogenicity	Focused on bacterial mechanisms; host immune response not assessed
Nemoto TK, Ohara Nemoto Y (7)	2021	Review	N/A	Review of dipeptidyl-peptidase function	Protein processing in P. gingivalis	Highlighted the role of dipeptidyl-peptidases in processing extracellular proteins, impacting bacterial virulence	Review article; experimental validation required
Miller DP, Scott DA (44)	2020	Review	N/A	Analysis of protein catabolism genes	Role in P. gingivalis metabolism and virulence	Identified inherently and conditionally essential genes for protein catabolism in P. gingivalis, highlighting targets for therapeutic intervention	Review article; lacks experimental validation
Ohara-Nemoto Y et al. (45)	2014	Experimental Study	Porphyromonas gingivalis	Identification of DPP-5	Enzyme characterization and function	Identified and characterized DPP-5 in P. gingivalis, contributing to understanding of bacterial protein processing and potential virulence	Focused on bacterial mechanisms; host interactions not explored
Shibata Y et al. (46)	2003	Experimental Study	Prevotella intermedia	Purification of DPP enzyme	Enzyme activity and characterization	Purified and partially characterized a DPP from P. intermedia, providing insight into its potential role in periodontal disease	Limited to *in vitro* findings; *in vivo* relevance not assessed
Grenier D et al. (47)	2001	Experimental Study	Porphyromonas gingivalis	Study of aminopeptidase activities	Enzyme activity and virulence	Demonstrated aminopeptidase activities in P. gingivalis, contributing to tissue destruction and virulence	Focused on enzymatic activity; host immune response not evaluated

- Host-derived DPP-4 activity: Multiple observational studies (24, 25, 33, 40) consistently reported elevated DPP-4 levels in saliva, gingival crevicular fluid, and gingival tissues in periodontitis patients. Longitudinal work showed that periodontal treatment reduced DPP-4-like protease activity, linking enzyme activity with disease progression.
- Bacterial proteases: Studies on *Porphyromonas gingivalis* and other pathogens (28, 34, 35, 37, 39, 43) demonstrated that bacterial DPPs degrade collagen and bioactive peptides, facilitate biofilm formation, and increase virulence. *In vitro* biofilm models (26, 27) showed that bacterial DPPs expand substrate specificity and degrade incretins, potentially impacting systemic glucose regulation.
- Therapeutic targeting: Monoclonal antibody inhibition of bacterial DPP-IV reduced *P. gingivalis* activity (30), whereas structural studies (28) provided potential frameworks for targeted drug design.
- Genomic and review insights (36, 44) underscored the multifactorial nature of disease, highlighting host genetic susceptibility and bacterial protein catabolism genes.

Together, these studies identify host–microbe enzymatic interactions as key drivers of periodontal tissue destruction and possible systemic effects.

### Therapeutic potential of GLP-1 receptor agonists in periodontal inflammation and regeneration

Twelve studies investigated GLP-1 receptor agonists and related incretin-based therapies for periodontal inflammation, osteoprotection, and regeneration (**Table 4**).

**Table 4:** Therapeutic potential of GLP-1 receptor agonists in periodontal inflammation and regeneration.

Author(s)	Year	Study design	Population	Intervention	Outcomes measured	Key findings	Limitations
Sawada N et al. (8)	2020	Experimental Study (Animal)	Rodent model	GLP-1 receptor agonist (Liraglutide)	Periodontal inflammation, bone loss	Liraglutide reduced periodontal inflammation and bone loss	Animal study; results may not directly translate to humans
Zhai S et al. (9)	2023	Experimental Study (Animal)	Zebrafish scale regeneration model	GLP-1 receptor activation	Osteoblast differentiation, bone formation	GLP-1 receptor promotes osteoblast differentiation and enhances bone formation	Animal model may not fully replicate human biology
Zhang Y et al. (10)	2020	*In vitro* and *in vivo* study	Periodontitis model (cell cultures and rodents)	Liraglutide	Bone destruction, inflammatory markers	Liraglutide reduces bone destruction and inflammation in periodontitis	Results in animal models may not be directly applicable to humans
Liang Q et al. (48)	2021	*In vitro* and *in vivo* study	Human periodontal ligament stem cells (PDLSCs) and animal model	Stromal cell-derived factor-1 and Exendin-4 co-therapy	Cell proliferation, migration, osteogenic differentiation, bone regeneration	Co-therapy enhanced proliferation, migration, and osteogenic differentiation of PDLSCs, promoting periodontal bone regeneration	Preclinical study; human clinical trials needed for validation
Pang Y et al. (49)	2019	*In vitro* study	Human periodontal ligament cells	Liraglutide	Cell proliferation, migration, osteogenic differentiation	Liraglutide promoted proliferation, migration, and osteogenic differentiation of PDL cells	*In vitro* results; clinical relevance requires further *in vivo* studies
Murai H et al. (50)	2024	Experimental Study	Porphyromonas gingivalis cultures	Curcumin	Bacterial growth, dipeptidyl peptidase activity	Curcumin inhibited DPP activity and growth of P. gingivalis, suggesting therapeutic potential	*In vitro* study; *in vivo* effects and clinical relevance need investigation
Moraes RM et al. (51)	2015	Animal Study	Rats with periodontitis	Exenatide and Sitagliptin	Inflammatory markers (IL-1β, MMP-9, NOS2), alveolar bone loss	Reduced inflammatory markers but did not decrease alveolar bone loss	Animal model; may not fully translate to human outcomes
Liu H et al. (52)	2019	*In vitro* study	Human periodontal ligament stem cells	Exendin-4	Wnt and NF-κB signaling, osteogenic differentiation	Exendin-4 regulated Wnt and NF-κB signaling, promoting osteogenic differentiation	*In vitro* model; clinical trials needed for confirmation
Guo Z et al. (53)	2018	*In vitro* study	Human periodontal ligament stem cells	Exendin-4	Cell proliferation, osteoblastic differentiation	Exendin-4 countered high glucose-induced inhibition, promoting proliferation and osteoblastic differentiation	*In vitro* model; clinical relevance needs to be explored
Eley BM, Cox SW (54)	1992	Correlational Study	Periodontitis patients	Measurement of protease activity in GCF	Clinical and radiological attachment loss	Significant correlation between GCF protease activity (including DPP IV) and periodontal attachment loss, indicating a role in disease severity	Correlational design; cannot establish causality
Qi J et al. (55)	2020	Experimental Study	Animal model	DPP-4 inhibitor administration	Tooth movement and root resorption	DPP-4 inhibitor reduced orthodontic tooth movement and root resorption, suggesting potential for therapeutic application	Animal study; human clinical relevance needs confirmation
Suzuki Y et al. (56)	2016	Experimental Study	Animal model of periodontitis	Glucose-dependent insulinotropic polypeptide (GIP)	Inflammation and periodontal tissue health	GIP exhibited anti-inflammatory effects in periodontitis, indicating a protective role and potential therapeutic use	Animal study; translation to human clinical application requires further investigation

- Anti-inflammatory and bone-protective effects: Animal studies consistently showed that GLP-1 RAs reduce periodontal inflammation and bone loss (8, 10). Moraes et al. (51) found reduced inflammatory mediators with exenatide and sitagliptin, though without significant bone preservation. Qi et al. (55) showed DPP-4 inhibitors reduced orthodontic root resorption.
- Osteogenic and regenerative capacity: *In vitro* and *in vivo* evidence demonstrated that GLP-1 RAs stimulate periodontal ligament cell proliferation, migration, and osteogenic differentiation (49, 52, 53). Zhai et al. (9) and Liang et al. (48) further supported regenerative potential, including synergistic effects with SDF-1.
- Complementary mechanisms: Suzuki et al. (56) reported that GIP exerted anti-inflammatory effects in periodontitis. Murai et al. (50) showed curcumin inhibited bacterial DPP activity, suggesting adjunctive antimicrobial potential.
- Clinical correlations: Classic work by Eley & Cox (41, 54) linked protease activity in gingival fluid with periodontal attachment loss, reinforcing the relevance of protease modulation.

Taken together, these findings suggest that GLP-1 RAs exert multifaceted benefits in periodontitis by reducing inflammation, protecting alveolar bone, enhancing osteogenesis, and potentially modulating microbial virulence.

## Discussion

This scoping review highlights a complex, interdependent network linking periodontitis, diabetes, and systemic metabolic dysfunction, with inflammation, oxidative stress, lipid metabolism, and microbial dysbiosis serving as central mediators. Elevated dipeptidyl peptidase-4 (DPP-4) activity and disturbances in glucoregulatory hormones—particularly in gingival environment—connect periodontal inflammation with impaired glycemic control. Evidence increasingly supports the role of GLP-1 and its analogues, such as exendin-4 and liraglutide, in not only improving glycemic outcomes but also enhancing bone regeneration, mitigating oxidative damage, and reducing local inflammatory responses. These effects are particularly relevant for diabetic patients, in whom lipotoxicity and advanced glycation end-products (AGEs) exacerbate periodontal tissue destruction, but may be attenuated by agents including metformin, omega-3 fatty acids, and GLP-1 analogues. Moreover, gut microbiota dysbiosis contributes to systemic inflammation and insulin resistance, while natural compounds such as resveratrol may help restore microbial and immune balance—underscoring the value of integrated therapeutic approaches.

Overall, the reviewed studies have reinforced the bidirectional relationship between periodontitis and cardiometabolic disorders. Periodontitis is more prevalent among individuals with obesity and type 2 diabetes, while also contributing to disease progression through chronic inflammation, oxidative stress, and dysregulation of the incretin axis. In severely obese populations, periodontitis correlates with elevated glucagon and GIP levels alongside reduced GLP-1, suggesting a mechanistic pathway that exacerbates glucose dysregulation. Notably, periodontal therapy has been shown to restore GLP-1 and GIP levels even in non-diabetic populations, although systemic markers such as hs-CRP often remain elevated in obese individuals, indicating an attenuated systemic response.

The therapeutic relevance of GLP-1 RAs is particularly compelling. Liraglutide demonstrated anti-inflammatory and bone-preserving effects in experimental periodontitis models through activation of the Nrf2/HO-1 oxidative stress pathway. GLP-1 receptor signaling also enhanced osteoblast differentiation in human dental pulp–derived stem cells via the LINC00968/miR-3658/Runx2 axis, sustaining osteogenesis even in hyperglycemic conditions. Similarly, exendin-4 reversed LPS-induced suppression of osteogenic differentiation in periodontal ligament stem cells (PDLSCs) by modulating NF-κB and Wnt/β-catenin signaling. Combined therapies, such as SDF-1 with Ex-4, further synergized to enhance PDLSC activity and bone regeneration *in vivo*, supporting the potential of stem cell–based regenerative approaches in periodontology.

The enzymatic role of DPPs, particularly DPP IV, emerges as another critical node at the intersection of microbial virulence and host systemic health. Elevated DPP IV activity in saliva and gingival crevicular fluid correlates with periodontitis severity and with the presence of *Porphyromonas gingivalis*. Since *P. gingivalis* relies heavily on DPP4, DPP5, DPP7, and DPP11 for nutrient acquisition in its asaccharolytic environment, inhibiting these enzymes disrupts its growth and pathogenicity. Strikingly, bacterial DPP IV mimics its human counterpart, degrading GLP-1 and thereby potentially worsening systemic insulin resistance. Host cells, including macrophages and fibroblasts, also upregulate DPP IV expression during inflammation, reinforcing its centrality at the host–pathogen interface. The structural similarities between bacterial and human DPP IV enzymes highlight opportunities to repurpose or redesign existing DPP-4 inhibitors for both systemic and periodontal applications.

Adjunctive approaches demonstrate potential. Curcumin, for instance, disrupts amino acid metabolism in *P. gingivalis* and inhibits DPP activity, causing nutrient deprivation stress. Human leukocyte elastase (HLE) has shown anti-inflammatory effects by downregulating CD40 on gingival fibroblasts, thereby impairing cytokine signaling central to periodontal tissue inflammation.

Together, these findings underscore the incretin axis and proteolytic enzyme systems as key regulatory nodes linking periodontal inflammation and systemic metabolic dysfunction. Targeting these pathways presents promising opportunities to develop dual-benefit therapies for both periodontal disease and cardiometabolic conditions.

## Future directions

### Animal studies and clinical translation

Future preclinical studies should use long-term and disease-complex models that better mimic chronic diabetes-associated periodontitis, including aged, obese, or genetically modified rodents. Studies of localized delivery systems for GLP-1 RA or DPP-4 inhibitors (e.g., biodegradable gels or microspheres) may clarify the feasibility of site-specific periodontal therapies with minimized systemic exposure. Moreover, animal models can clarify the impact of incretin-based therapies on the oral microbiome, oxidative stress, and immune-cell dynamics (e.g., M1/M2 macrophage polarization). Gene knockout or CRISPR-based modulation of bacterial and host DPP activity could further reveal mechanistic drivers of disease progression and resolution.

### Clinical trials

Translation to human trials is the next critical step. Early-phase studies should assess GLP-1 receptor agonists and DPP-4 inhibitors as adjuncts to conventional periodontal therapy, particularly in patients with metabolic comorbidities. Non-diabetic or prediabetic populations with moderate-to-severe periodontitis may be ideal initial cohorts. Primary endpoints may include clinical attachment gain, inflammatory biomarkers, and systemic measures such as GLP-1 levels, HbA1c, and lipid profiles.

### Randomized controlled trials

Comparisons of administration routes (oral, injectable, and localized delivery) and patient subgroups (e.g., obese vs. non-obese) will provide valuable insight into personalized therapy. Incorporating microbiome profiling and biomarker analysis may further reveal systemic and microbial shifts associated with treatment. Such translational trials will be essential to validate the dual periodontal and metabolic benefits of incretin-based therapies and protease inhibitors.

## Conclusion

In summary, preclinical and observational evidence provides a strong rationale for exploring GLP-1 receptor agonists and DPP-4 inhibitors as novel adjunctive therapies in periodontology. Although preliminary data are promising, well-controlled human trials—conducted with careful ethical oversight and interdisciplinary collaboration—will be key to advancing these therapies into clinical practice. By targeting shared pathways of metabolic and periodontal dysfunction, incretin-based therapies hold potential to transform periodontitis management within the broader landscape of cardiometabolic health.
